# Comprehensive insights into phosphorus solubility and organic matter’s impact on black phosphate leaching

**DOI:** 10.1038/s41598-024-69399-z

**Published:** 2024-08-19

**Authors:** Houda A. Khedr, Mohamed O. Ebraheem, Ahmed M. Zayed

**Affiliations:** 1 Geology Department, Faculty of Science, New Valley University, New Valley, Egypt; 2 Applied Mineralogy and Water Research Lab (AMWRL), Geology Department, Faculty of Science, Beni-Suef University, Beni Suef, 62521 Egypt

**Keywords:** Black phosphate, Organic matter, Calcination, Hydrogen peroxide, Acid leaching, Nano scale, Solid Earth sciences, Chemistry, Nanoscience and technology

## Abstract

The current study introduces groundbreaking insights into how organic matter (OM) of the black phosphate (RB-Ph) uniquely influences phosphorus (P) solubility during acetic acid (AA) leaching, expanding our understanding in this crucial area. To highlight such role, the OM of the RB-Ph was treated separately by different procedures including calcination at 550 ℃/4 h (CB-Ph), 30% hydrogen peroxide (HB-Ph) and intensive grinding to nano-sizes (NB-Ph). The mineralogical, chemical and morphological characteristics of phosphatic and non-phosphatic components of these phosphatic materials were carefully examined pre- and post-treatment via different techniques. The P dissolution of the precursor RB-Ph and its modified derivatives all over the applied experimental parameters traced the following trend: NB-Ph > RB-Ph > CB-Ph > HB-Ph. Intensive grinding to nanoscale resulted in amorphous components with conspicuous OM content (TOC, 0.410%), significantly enhanced P dissolution rate of NB-Ph (730–980 ppm), despite the noticeable reduction in its P_2_O_5_ content to 22.34 wt.%. The precursor RB-Ph, thanks to its high OM content (TOC, 0.543%), also displayed a sufficient P dissolution rate (470–750 ppm) compared to the two other modified derivatives, CB-Ph (410–700 ppm) and HB-Ph (130–610 ppm). Such deep and conspicuous impact of OM on P solubility can be tied to their decomposition, releasing not only organic acids but also the adsorbed P by the OM’s surficial binding sites to the solution. Finally, the optimum conditions of P leaching were attained at 2:1 acid/solid (w/w) ratio and 2 h of retention time of all investigated samples.

## Introduction

Chemical phosphatic fertilizers, triple super phosphate (TSP), mono-and diammonium phosphate (MAP and DAP), and ammonium polyphosphate liquid (APP), are extensively used to achieve high crop yields. However, these fertilizers contain highly soluble forms of phosphorus, P, that can easily become insoluble and precipitate^1^, leading to extreme and repeated application of phosphorus fertilizer. The latter can cause raised levels of P in surface water due to runoff loss from agricultural land. This P flux is a significant cause of eutrophication in surface water bodies as lakes and coastal marine environments^2^.

Phosphate fertilizer production accounts for approximately 95% of the world’s annual production of phosphate rocks (PRs), which extends to around 150 million tons^3^. Primary environments where phosphate minerals are found include igneous and metamorphic environments, as carbonatites; fluorapatite (Ca_10_(PO_4_)_6_F_2_) is prevalent. On the other hand, hydroxyapatite (Ca_10_(PO_4_)_6_(OH)_2_), is not only found in igneous and metamorphic environments, but also in biogenic deposits such as bone deposits. Lastly, carbonate hydroxyapatites (Ca_10_(PO_4_, CO_3_)_6_(OH)_2_) are mainly found in islands and caves as part of bird and bat excrements or guano^4^. Sedimentary marine deposits account for 75% of the world’s phosphate resources, while igneous, metamorphic, and weathered deposits make up 15–20%, and biogenic accumulations share is only 2–3%^5^. The majority of the principal phosphate minerals found in these sedimentary PRs is carbonate fluorapatite, which is typical of secondary origin. This carbonate fluorapatite contains more than 1% F and significant amounts of CO_2_^6^. Regarding ore grade, phosphate ores are categorized into three groups based on their P_2_O_5_ concentration: low-grade (12–16%), medium-grade (17–25%) and high-grade ores (26–35%)^7,8^.

Phosphorous (P) is an important nutrient for ecosystem structure, and cellular processes, including maintenance of membrane structures, synthesis of biomolecules and formation of high-energy molecules. It also helps in cell division, enzyme activation/inactivation and carbohydrate metabolism^9,10^. Thus, scientists tried to increase the solubility of P of the PRs through the reduction of their associated gangues such as carbonates, organic matter (OM) and clays to increase the potentiality of using PRs as efficient fertilizer. This can be conducted through several procedures such as washing to eliminate clays, calcination to dispose carbonates or organic matters^11^. Also, the oxidation by H_2_O_2_ is one of the effective methods for OM reduction^12^ as it can create more reactive surfaces or modified mineral structures. Moreover, the intensive grinding to nano sizes may induce the decomposition/oxidation of the associating OM as volatile substances, altering their chemical composition (chemical bond breakage)^13^.

Black phosphate (BP), the core of the current study, is a definite type of sedimentary phosphate that is rich in OM. Ores of this type are generally beneficiated by heating up to about 800 ℃^14^. This type of calcination burns organic material and residual organic carbon without significantly affecting the superior qualities of sedimentary phosphates such as solubility and reactivity. Furthermore, because of low calcination temperature, the reduction of calcium sulfate, present in ore, to corrosive calcium sulfide by the organic matter is minimized. During the burning of OM, the organic carbon must be decreased < 0.3% to minimize the gassing in the wet phosphoric acid processing. As well the apatite CO_2_ must be maintained at a level close to 2% to allow good reactivity of calcined product^3,15^. Although the potential of the BP as a phosphorus resource, the high OM content can affect its applicability and pose challenges in terms of processing, product quality and environmental impact. OM can interfere with nutrient uptake by plants or contribute to nutrient imbalances in the soil^16,17^. During the decomposition process of OM, microorganisms utilize nitrogen (N) and other nutrients for their own growth and reproduction. This can temporarily immobilize nutrients, making them less available for plant uptake. This phenomenon is known as nutrient tie-up or immobilization. Moreover, OM can form stable complexes with certain nutrients, particularly metals such as iron and aluminum; these complexes can reduce the availability of these nutrients for plant uptake. Therefore, the amount of OM is a sensitive component of BP, affecting its quality and applicability.

While extensive research has been conducted on general phosphorus solubility and treatment methods^18,19^, there are significant gaps in understanding how different OM treatments specifically affect P solubility. Previous studies have established that OM can either enhance or inhibit P solubility by influencing mineral dissolution and chelation processes^20–22^. However, these studies often focus on general OM interactions without delving into the specific effects of different OM treatments. Furthermore, detailed impact of various OM treatments on the solubility of P, particularly in black phosphate, remains underexplored. Most research does not differentiate the effects of physical, chemical, and mechanical OM treatments on P release dynamics^23,24^. Yet, the interplay between these treatments and OM content in modulating P solubility is not well understood. This is correlated with the lack of comprehensive studies that systematically compare the effects of these treatments on the behavior of OM and its subsequent impact on P solubility. Also, comparative studies that address the variability in OM treatment effects across diverse black phosphate deposits are sparse, leaving a gap in understanding the generalizability of treatment methods^25,26^. So, a clear understanding of how treatment-induced changes in OM content and form affect P dissolution is not provided in the current literature work. Research has shown that the effectiveness of P solubilization methods can vary significantly based on the type of phosphate deposit. However, there is limited knowledge on how treatment-induced OM modifications influence P solubility across different black phosphate deposits. Therefore, to fill these gaps the current study aims to: (1) Systematically investigate the impact of different processing protocols, calcination at 550 ℃/4 h, H_2_O_2_ (30%) treatment and intensive grinding to nano-sizes, upon OM content, mineral composition, structure and reactivity of phosphatic and non-phosphatic components BP deposits of Abu Tartur area; (2) The potential role of OM upon the P solubility from the raw and modified phosphorite samples, using acetic acid at different experimental parameters (i.e. acid concentration and contact time).

## Materials and methods

### Materials and chemicals

High-grade, non-oxidized black phosphorite deposit with high organic matter content was obtained from Abu Tartur plateau in the Liffiyia–Maghrabi sector, about 50 km to the west of Kharga Oasis, Western Desert, Egypt: latitude 25°25′34′′N and longitude 30°05′08′′E. Hydrogen peroxide (H_2_O_2_, 30%) was used as an oxidizing agent for the organic matters associated with the collected non-oxidized phosphorite technical sample. Whereas organic acetic acid was utilized as a leaching agent, and distilled water (DW) was the washing and liquefying solution.

### Samples preparation

#### Raw black phosphate (RB-Ph) preparation

Using a jaw crusher, the collected non-oxidized high-grade phosphate rock samples were crushed separately into size fractions of < 4 mm. Then these fractions were handed over to disc mill to obtain fractions ≥ 100 µm. The ground samples were thoroughly mixed till complete homogenization. Afterwards, the homogenous sample was quartered several times to get a representative sample. This phosphorite rock sample (PR) was washed with distilled water and dried in an electric oven at 70 ℃ overnight. About 500 g of the dried PR sample was reground in a laboratory ball mill (RETSCH PM 100/Retsch GmbH, Hann, Germany) and sieved to get size fractions ≤ 100 μm that was divided into four portions each of them is about 100 g in weight. The 1st portion was Packed and labeled as RB-Ph (raw black phosphate).

#### Nano-size black phosphate (NB-Ph) preparation

To prepare nano-size black phosphate** (**NB-Ph), the 2nd portion of the prepared PR was further reground several times using a high-density ball mill to get nano-size fractions (≤ 200 nm). Such portion was labeled as NB-Ph (nano-size black phosphate).

#### H_2_O_2_ treated black phosphate (HB-Ph) preparation

To get rid of the associating OM the PR via chemical protocol, hydrogen peroxide (30%) was employed as an effective oxidizing agent. Therefore, the 3rd portion of the prepared PR sample (about 100 g) was added to a beaker containing one liter of H_2_O_2_ (30%). Then the produced mixture was heated at 50 ℃/12 h with continuous vigorous stirring. The solid phase of this mixture was allowed to settle down before liquid decantation and the rejuvenation of the H_2_O_2_ solution for the next cycle. Such steps were repeated till effervescence cessation. Then the solid–liquid phases were separated by centrifuging at 10,000 rpm/15 m. Additionally, the obtained solid was washed carefully with DW several times and oven dried at 70 ℃ overnight before gentle grinding in a gate mortar and packing as HB-Ph (H_2_O_2_ treated black phosphate) for further application.

#### Calcinated black phosphate (CB-Ph) preparation

Similarly, to oxidize the OM, the 4th portion of the prepared PR sample (about 100 g) was calcinated in a muffle furnace at 550 ℃/4 h) before re-grinding gently in agate mortar and packing as CB-Ph (calcinated black phosphate) for further utilization.

### Characterization of the prepared samples

The prepared phosphorite samples were characterized using various techniques. The mineralogical composition of the RB-Ph sample was identified using optical microscopy (Nikon Eclipse LV 100 POL, Japan) in both plane-polarized (PPL) and crossed-polarized Light (XPL). However, the associating opaque components were elaborated using the same microscope, but in reflected mode. Moreover, the chemical composition and mineralogical phases of all investigated samples were identified by XRF and XRD (at a scanning speed of 5°/min in the 2θ range of 5°–80°) techniques, using Philips X-ray Fluorescence analyzer (model PW/2404) and Philips diffractometer (model APD-3720), orderly. Similarly, FT-IR analyses were conducted using a Bruker FT-IR-2000 Spectrometer in transmittance mode between 400 cm^−1^ and 4000 cm^−1^ with a nominal mode of reflection at a 4 cm^−1^ resolution to determine the functional groups of the prepared phosphorite samples. Additionally, scanning electron microscopy, SEM (JSM-6700F, JEOL, Tokyo, Japan, beam energy: 20–30 kV, working distance: 11.1–12.2 mm) equipped with an energy-dispersive X-ray spectrometer (EDX), was used to elucidate the morphological/chemical characteristics of the prepared samples after ascending on stubs and coating with gold via a gold-coating device (JEOL-JSM-420, Japan). Furthermore, the BET surface area (S_BET_, m^2^/g), total pore volume (V_t_, cm^3^/g), and average pore diameter (Dp, nm) of all addressed samples (RB-Ph, CB-Ph, HB-Ph, and NB-Ph), were determined using surface area analyzer (Quantachrome/Nova 2000). As well, the total organic carbon (TOC), total carbon (TC) and total sulfur (TS) of the raw sample and its derivatives, were investigated via Leco Sc 623 carbon analyzer device after digestion with a hot HCL (10%) to get rid of the carbonate impurities.

### Phosphorus solubility experiments using acetic acid as a leaching agent

The acetic acid (AA) leaching experiments were meticulously designed to evaluate the solubility of phosphorus (P) in black phosphate (RB-Ph) and its modified derivatives (CB-Ph, HB-Ph, and NB-Ph) under varying experimental conditions. Therefore, a series of controlled leaching experiments were conducted using an acetic acid solution, chosen for its mild acidity, which simulates organic acid interactions in natural soil environments and effectively dissolves mineral-bound P. The applied liquid/solid ratios (acid/phosphorite) as displayed in Table 1, were selected to ensure sufficient interaction between the employed phosphorite samples and the AA leaching agent, facilitating the dissolution of P. As well, the leaching durations (Table 1) were selected to provide a balance between sufficient reaction time and practical experimental efficiency. The temperature was kept constant at 25 ℃ to replicate ambient conditions and avoid thermal decomposition of sensitive organic matter. Stirring at 200 rpm ensured uniform dispersion of the sample in the solution, enhancing the contact between acetic acid and the phosphate particles. These conditions were selected to optimize P solubility and to closely mimic natural leaching processes, allowing for meaningful comparison across different OM treatment methods (calcination, H_2_O_2_ treatment, and intensive grinding). This setup aimed to provide consistent and reproducible results, reflecting the realistic behavior of black phosphate in agricultural and environmental contexts. Hence to compare the P solubility of the prepared samples (RB-Ph, CB-Ph, HB-Ph, and NB-Ph) using liquified acetic acid (98%) with specified volume of DW (25 ml) at room temperature, the following experimental parameters were carefully investigated: effect of acid concentration and effect of applied contact time. The prevailing experimental conditions are compiled in Table 1. Similarly, regeneration studies using acid to phosphorite material ratio of 2:1 were conducted to determine the number of cycles by which P can be leached from each of the addressed samples separately. Generally, to estimate the amount of dissolved P during the leaching process, the solid–liquid phases were separated using a digital centrifuge at 10,000 rpm for 15 min. Furthermore, the liberated P in the liquid phase for each sample was estimated by inductively coupled plasma optical emission spectrometry (ICP OES, Optima 2000 DV). For regeneration studies, the separated solid phases were dried at 105 ℃ overnight before undergoing several acid-leaching repeated cycles till the consumption of soluble P in the utilized phosphorite sample. Prior to each leaching cycle, the solid fractions were reweighed to determine how much mass was lost during the previous cycle and to maintain the 2:1 ratio (Table 1).

**Table 1:** Applied experimental parameters and prevailing conditions during the phosphorus (P) leaching experiments from the prepared phosphorite samples by acetic acid (AA).

Investigated parameter	Prevailing conditions					The other parameters
Acid: phosphorite samples concentration (g)	(0.5:1) 0.75: 1.5 g	(1:1) 1.5:1.5 g	(2:1) 3:1.5 g	(3:1) 4.5:1.5 g	(4:1) 6:1.5 g	25 ml DW, 200 rpm/2 h (speed/agitation time)
Contact time (h)	½	1	2	4	6	AA/PS (2:1 ratio: 3 g acetic acid: 1.5 g phosphorite sample), 25 ml DW, 200 rpm (speed)
Regeneration studies of phosphorite samples (g)	Cycle 1 (10:5 g)	Cycle 2 (8:4 g)	Cycle 3 (6:3 g)	Cycle 4 (4:2 g)	Cycle 5 (2:1 g)	AA/PS (2:1) ratio, 25 DW, 200 rpm/2 h (speed/agitation time)

## Results and discussion

### Field characteristics

**Figure 1:**
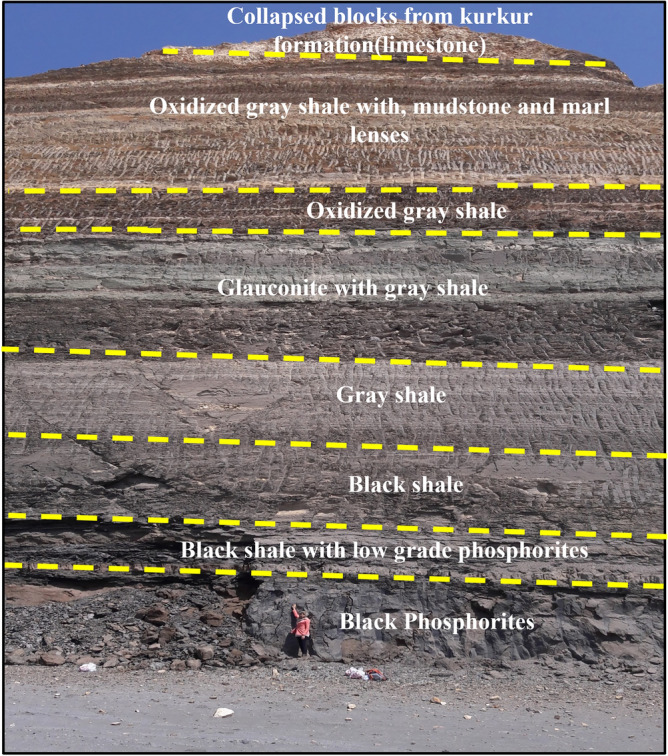
A field photograph showing a stratigraphic section of Duwi formation at the western region of the Liffyia–Maghrabi sector in Abu-Tartur mine.

The Duwi formation, which dates from the Campanian–Maastrichtian period^27^ and is part of the Abu Tartur plateau, contains the unoxidized phosphorites (black to greyish–black in color) used in this current study. Generally, the phosphorite deposits of Abu Tartur plateau are thought to be the highest grade in Egypt and have a reserve of 1 billion metric tonnes including both oxidized and non-oxidized varieties with an average of P_2_O_5_ of about 25%^12,28^. Duwi Formation in the Abu-Tartur area serves as a notable instance of a significant marine transgression occurrence in Egypt^29,30^. It consists of phosphorite accumulations, black shale and glauconite deposits (Fig. 1)^12^. The lower section contains phosphorite sand and thin black shale layers^31^. While the upper part is made up of thick brownish to dark-grey flaky shale layers with occasional presence of mudstone and marl lenses, as well as glauconite deposits from the Campanian–Maastrichtian period (middle layer)^32,33^. The cap on the plateau surface consists of limestone of the Kurkur formation^34^.

### Characterization of prepared samples

#### Petrographical characteristics

**Figure 2:**
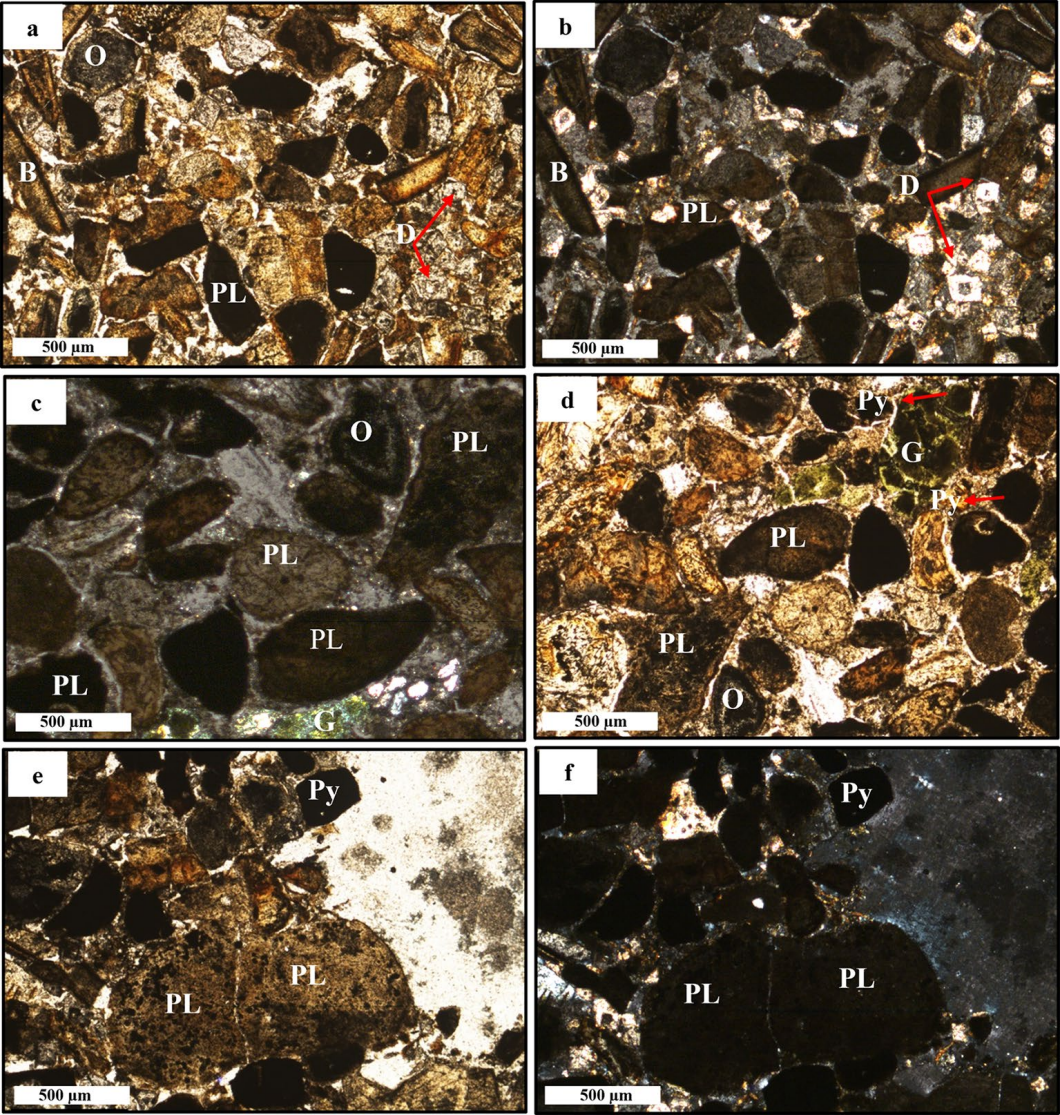
Petrographical images of black phosphate (RB-Ph) in PPL (**a**,**d**,**e**) and XPL (**b**,**c**,**f**) visions: peloid grains (PL), dolomite rhombs (D), ooids (O), fish bones (B), pyrite crystals (Py) and glauconite (G).

**Figure 3:**
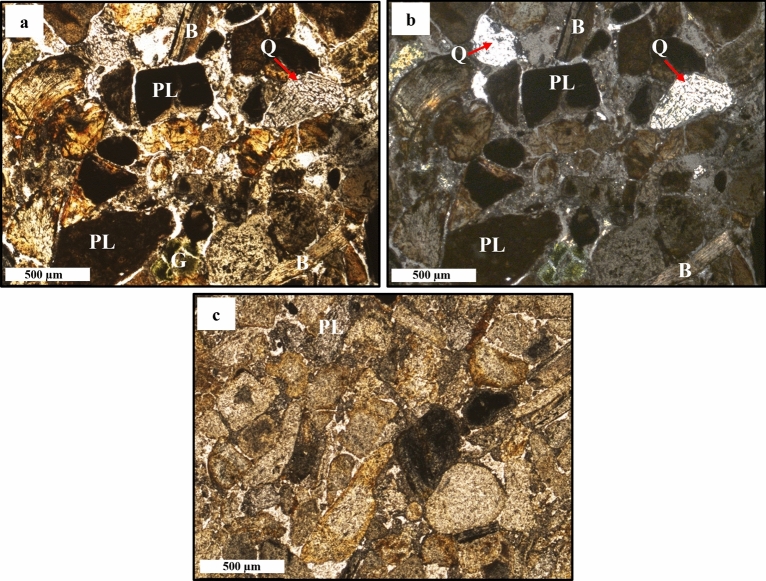
Petrographic images of black phosphate (RB-Ph) in PPL (**a**,**c**) and XPL (**b**) visions: peloid grains (PL), fish bones (B), glauconite (G) and quartz (Q).

**Figure 4:**
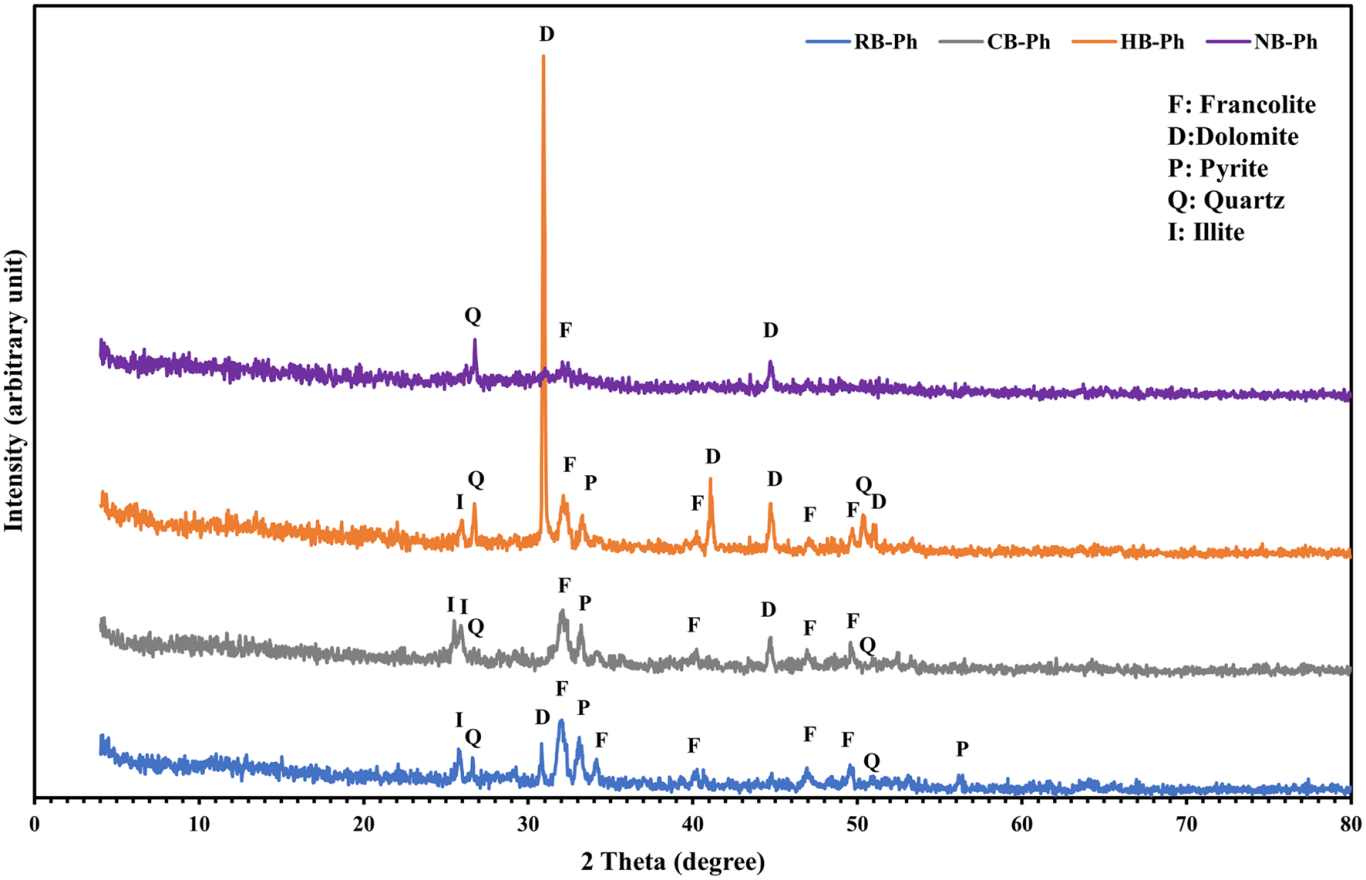
XRD patterns of the precursor black phosphate (RB-Ph) in comparison with its modified derivatives, calcination at 550 ℃/4 h (CB-Ph), 30% hydrogen peroxide (HB-Ph) and intensive grinding to nano-sizes (NB-Ph).

Transmitted light microscope equipped with Nikon camera was utilized to examine and capture images of the pristine, non-oxidized phosphorite deposits (RB-Ph) in both optical visions, in PPL and XPL (Fig. 2a–f and Fig. 3a–c). These deposits are composed mainly of phosphatic (mudclasts and bioclasts) and non-phosphatic grains^35^, as demonstrated by high magnifications. Phosphatic pellets, also known as mudclasts, exhibit a range of shapes and sizes, from angular to sub-rounded. When viewed in PPL (Fig. 2a, d and Fig. 3a, c), they are typically homogeneous and lack any discernible structure, although occasional ooids may be present (Fig. 2c, d). These ooids form through the growth and crystallization of micrometric phosphate layers^32,36,37^. Additionally, the phosphatic grains’ colour varies from dark to light brown, which is probably related to the variations in the content of organic matter and/or iron oxide. But sometimes they appear isotropic between Crossed Nicols (Fig. 2b, c, f), aligning with previous investigations^29^. In contrast, fish bones and shark teeth (bioclasts) are less common than phosphatic mudclasts in the investigated samples and are naturally colourless, elongated, and angular to sub-angular in shape (Fig. 2a, b and Fig. 4a, b). Furthermore, they exhibit low birefringence and display grey interference colours of the first order, as well as lamellar twinning or undulatory extinction as was described before by several studies^38,39^. The non-phosphatic component is distinguished by the presence of undesirable impurities such as abundant carbonate minerals, dolomite (Fig. 2a, b), and detrital quartz (Fig. 3a, b). Also, pyrite (Fig. 2d–f) was frequently observed, along with glauconite (Fig. 2c, d), matching with previous investigations^28,40,41^.

#### Geochemical characteristics

The chemical composition of the studied samples (RB-Ph, CB-Ph, HB-Ph, and NB-Ph) is depicted in Table 2. It was revealed that P_2_O_5_, CaO, SiO_2_, SO_3_ and Fe_2_O_3_ are the main components of these samples. According to the P_2_O_5_ (30.5 wt.%) content, the pristine un-oxidized phosphorite deposits (RB-Ph) can be classified as high-grade deposits^42,43^. However, it was noted that many of the major oxides in the treated samples (CB-Ph, HB-Ph, and NB-Ph) demonstrate distinct variations, indicating the conspicuous impact of these processes upon their chemical composition with respect to the raw one. The high reduction of CaO, P_2_O_5_, Fe_2_O_3_, F and SO_3_ contents of HB-Ph into 29.74, 21.6, 2.96, 1.31 and 2.27 wt.%, respectively, confirms the destructive impact of this acidic reagent not only upon the organic matter (i.e., reduction of organic sulfur that was expressed in the abatement of SO_3_ wt.%) of the pristine RB-Ph sample, but also upon its phosphatic (CaO, P_2_O_5_, and F wt.% demolition) and pyrite minerals (Fe_2_O_3_ wt.% drop)^44^. Conversely, the noticeable enrichment of SiO_2_, Al_2_O_3_, MgO, MnO, K_2_O and LOI after H_2_O_2_ treatment (15.90, 3.62, 1.96, 0.40, 0.15 and 19.37 wt.%) compared to the pristine sample could be ascribed to the concentration of clays, quartz, and dolomite in HB-Ph sample (Table 2). Similarly, the impact of intensive grinding to nano-size (NB-Ph) followed the same trend of reduction and enrichment in the chemical composition like the H_2_O_2_ treated sample (HB-Ph), but with little magnitude, if compared with the pristine one (Table 2). This was assured by the reduction of CaO, P_2_O_5_, Fe_2_O_3_, F and SO_3_ contents (34.92, 22.34, 3.36, 1.12 and 7.54 wt.%, respectively) and the enrichment of SiO_2_, Al_2_O_3_, MgO, MnO, K_2_O and LOI contents (9.1, 1.76, 1.92, 0.33, 0.18 and 16.47 wt.%, orderly) after grinding compared to RB-Ph sample (Table 2). The intensive leaching ability of the H_2_O_2_ that contributed to an increase in the solubility of CaO, P_2_O_5_, Fe_2_O_3_, F and SO_3_ contents in HB-Ph sample explains the invasive impact of such oxidizing agent upon the whole chemical composition of the HB-Ph sample compared to the grinding process at the NB-Ph one (Table 2)^45^. With respect to the calcination treatment process, approximately a slight variation in the chemical composition of the CB-Ph sample was observed in correlation with RB-Ph (Table 2). However, the adequate improvement of the SO_3_ content (13.74 wt.%) in the CB-Ph compared to RB-Ph (11.88 wt.%), can be ascribed to the partial oxidization of pyrite (i.e., the oxidation of sulfide into sulphate), compensating the loss of organic sulfur during the oxidation of organic matter by calcination^46^. On the contrary, the reduction in LO I from 9.23 wt.% in the RB-Ph to 7.75 wt.% in CB-Ph, can be ascribed to the oxidation of the organic matter and clay mineral dehydration of the pristine sample by calcination, aligning with the previously discussed petrographical investigations.

**Table 2:** Chemical composition of the precursor black phosphate (RB-Ph) in comparison with its modified derivatives, CB-Ph (calcinated at 550 ℃/4 h), HB-Ph (30% hydrogen peroxide treated) and NB-Ph (intensively ground to nano-sizes).

Major elements	RB-Ph (wt.%)	CB-Ph (wt.%)	HB-Ph (wt.%)	NB-Ph (wt.%)
SiO_2_	3.19	3.26	15.90	9.10
TiO_2_	0.03	0.04	0.13	0.11
Al_2_O_3_	0.64	0.61	3.62	1.76
Fe_2_O_3_	4.61	4.51	2.96	3.36
MnO	0.22	0.21	0.40	0.33
MgO	0.63	0.80	1.96	1.92
CaO	36.20	35.17	29.74	34.92
Na_2_O	0.53	0.56	0.27	0.26
K_2_O	0.05	0.06	0.15	0.18
P_2_O_5_	30.51	30.36	21.60	22.34
SO_3_	11.88	13.74	2.27	7.54
F	1.80	1.83	1.31	1.12
CL	0.04	0.64	0.03	0.05
LOI	9.23	7.75	19.37	16.47
Total	99.55	99.54	99.71	99.46

#### Mineralogical characteristics

The XRD patterns of the investigated phosphorites, RB-Ph, CB-Ph, HB-Ph, and NB-Ph, are depicted in Fig. 4. Concerning the XRD pattern of the pristine sample, RB-Ph, the phosphatic phase is francolite. Whereas the other associating non-phosphatic components include dolomite, pyrite, illite and quartz that were expressed by peaks of various intensities. Also, the associating organic matter were reflected by amorphous noise in the XRD pattern^47^. Francolite, dolomite and pyrite were reflected by intensive peaks at 2θ = 31.9°, 30.7°, and 33° with d-spacings of 2.8, 2.7, and 2.9°A, orderly. Meanwhile, the minor peaks with less intensities at 2θ = 25.7°, 26.7°, and 56.1° could be attributed to illite, detrital quartz and pyrite minerals, respectively^48,49^. After calcination at 550 ℃/4 h, the main peak of francolite was slightly reduced in intensity and shifted from 2θ = 31.9° to 32° in CB-Ph sample. As well, the other non-phosphatic phases, witnessed an observable reduction in their intensities (illite & pyrite), except for dolomite peak at 2θ ≈ 44.9° which witnessed an appreciable improvement in intensity with calcination. But with H_2_O_2_ treatment (HB-Ph), the phosphatic components displayed a noticeable reduction in their intensities, giving the space for dolomite to occupy the main peaks (Fig. 4). This is matching with XRF data that confirmed a remarkable increase in MgO wt.% gained from the dominating dolomite^50^. While pyrite continued demolition as a consequence of oxidation by H_2_O_2_. Also, the perceptible presence of quartz 2θ = 26.7° and 50.3° confirms that H_2_O_2_ does not affect quartz^51^. Therefore, the domination of non-phosphatic minerals at the expense of phosphatic ones, confirms XRF results concerning the observable reduction in P_2_O_5_ content of HB-Ph. Additionally, the intensive grinding to nano size (NB-Ph), contributed to an observable destruction, not completely, of both phosphatic and non-phosphatic phases^49^ (Fig. 4). This was assured by the domination of amorphous noise, except for some quartz and dolomite relics.

#### Spectral characteristics

**Figure 5:**
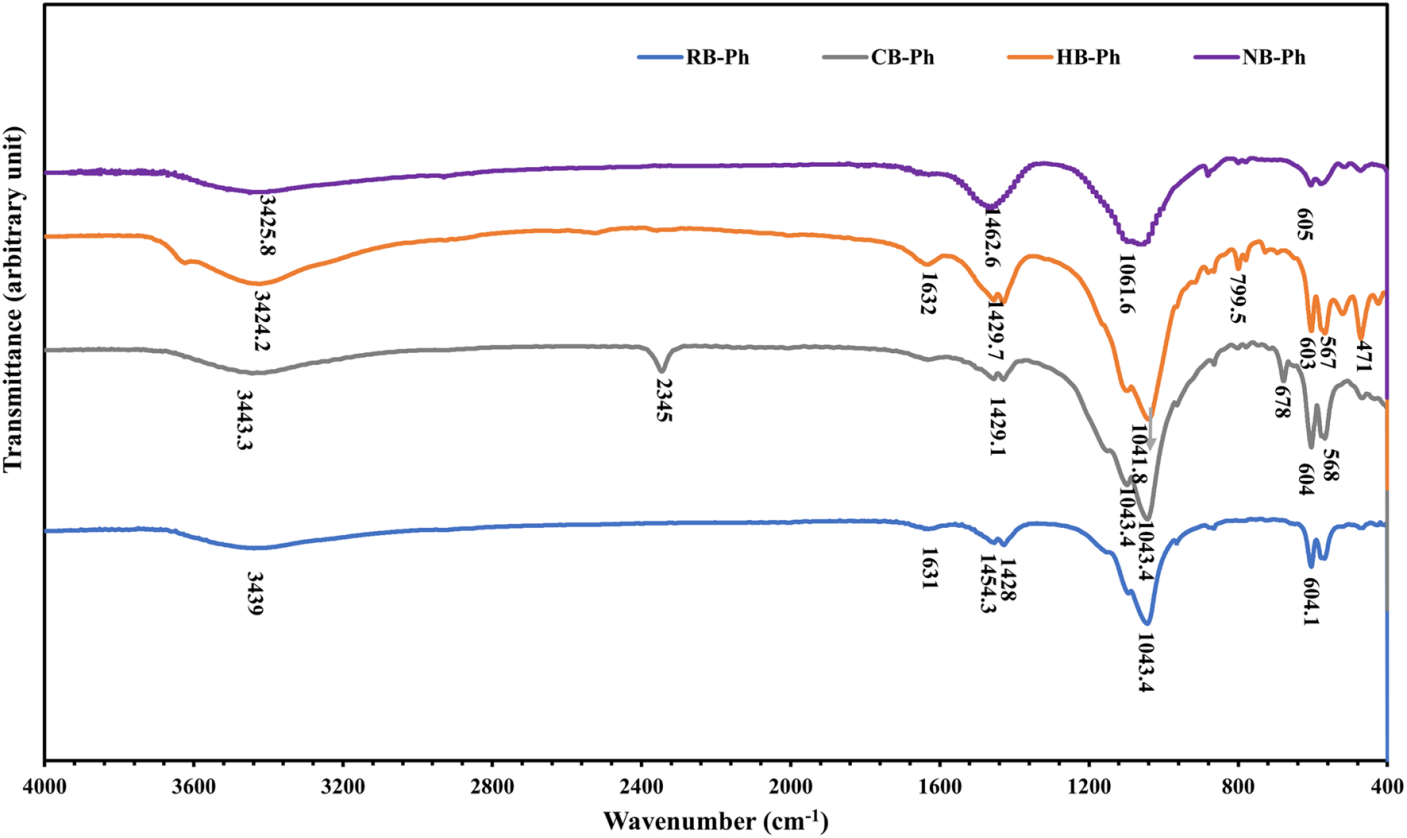
FT-IR spectra of the precursor black phosphate (RB-Ph) in comparison with its modified derivatives, calcination at 550 ℃/4 h (CB-Ph), 30% hydrogen peroxide (HB-Ph) and intensive grinding to nano-sizes (NB-Ph).

The spectra of the main functional groups of the investigated phosphorites (RB-Ph, CB-Ph, HB-Ph and NB-Ph) are displayed in Fig. 5. The main functional groups of the pristine phosphorite sample (RB-Ph) emerged at the following absorption bands: 3439, 1631, 1454.3, 1428, 1043.4, 604.1, 568.8, 467.1 cm^−1^. Some of these bands reflect the overlapped signatures of both organic and inorganic components of the RB-Ph sample: 3439, 1631 and 604.1 cm^−1^, while the other bands can be correlated to the inorganic components only. The 3439 cm^−1^ band can be linked with the stretching vibrations of either hydroxyl (O–H) or N–H groups^52–56^. The latter group expresses the signature of the organic matter of the pristine sample. Also, the weak band at 1631 cm^−1^, not only was attributed to the bending mode of water “δ (H_2_O)”^57,58^, but also falls in the range of C = C (alkene) stretching vibrations^59–62^. The latter is typically associated with the presence of conjugated double bonds of the aromatic compounds or unsaturated moieties of the associating organic matter^59,63^. Similarly, it is possible to consider multiple options for the assignment at 1454.3 and 1428 cm^−1^ bands^64^. Where, both the anti-symmetrical bending vibration (ν_3_) of the carbonate (CO_3_)^2−^ group and C-H bending vibrations of the aliphatic hydrocarbon chains as those found in alkanes, could contribute to these absorption bands in the infrared spectrum^65–69^. Furthermore, the 604.1 cm^−1^ band, which was attributed to the bending or rocking vibrations of the (PO4)^3−^ group of the inorganic phosphates, may overlap with other vibrations from organic phosphate^66,70^. On the contrary, the 568.8 and 467.1 cm^−1^ bands are typically associated with the bending or rocking vibrations of the (PO_4_)^3−^ group^71,72^, which is commonly found in inorganic phosphates rather than organic phosphates. Regarding the absorption band at 1043.4 cm^−1^, in the RB-Ph spectra, it could be correlated with the stretching vibrations of Si–O bond of the inorganic components only (i.e., silica, quartz or silicate minerals, illite), matching with XRD and petrographical data^73^. With calcination at 550 ℃ /4 h, it was observed that the spectra of the CB-Ph sample witnessed the emergence of a new absorption band at 2345 cm^−1^ that was correlated to the asymmetric stretching vibration of the carbon–oxygen double bond in CO_2_^74–78^. The presence of trapped carbon dioxide within the sample pores could be ascribed to the thermal decomposition/oxidation of carbonaceous materials in OM of RB-ph during the calcination process, releasing some CO_2_. Also, the emergence of a new band at 678 cm^−1^ after calcination could be ascribed to the stretching mode of C–Cl group of the chlorinated organic residues. This was assured by the high Cl ratio (0.64 wt.%) that amplified about 16-fold compared to the pristine sample, 0.04 wt.% (Table 2). The calcination process likely led to the thermal decomposition or oxidation of carbonaceous materials in OM, resulting in the release and subsequent trapping of chlorinated compounds within the sample. Similarly, the amplification of the (PO_4_)^3−^ and Si–O bands at 604, 568 and 1043 cm^−1^, orderly, is likely associated with the decomposition/oxidation of the associating OM in the pristine sample with calcination. It also indicates the enhanced presence of these functional groups, aligning well with XRD and XRF data (Fig. 4 & Table 2). Similarly, the chemical treatment of RB-Ph with H_2_O_2_ (30%), contributed to an improvement in the intensity of the (CO_3_)^2−^ group at 1429.7 cm^−1^ in accordance with the oxidation of OM and some of the phosphatic components. This drove toward the domination of dolomite as displayed in XRD and XRF (LOI = 19.37 and MgO = 1.96 wt. %) (Fig. 4 & Table 2, orderly). This could be assigned to the specific reactivity of dolomite towards H_2_O_2_, which allowed it to withstand the treatment while other minerals or OM were affected. Additionally, the approximate strengthening of the (PO_4_)^3−^ and Si–O bands at 603, 567, 471 and 1041.8 cm^−1^, orderly, in HB-Ph sample, could be confined with the OM/some phosphatic components oxidation via H_2_O_2_ as a sign of concentration of both phosphate and silicate species, aligning well with XRD data. The presence of significant amounts of SiO_2_ (15.9 wt.%) and Al_2_O_3_ (3.62 wt.%), further confirms the increased concentration of silicate species in the HB-Ph sample (Table 2). This was also assured by the noticeable presence of Si–O–Si group in bending mode at 799.5 cm^−1^ of the silicate components^79^. On the contrary, the distinguishing functional groups of the intensively grounded NB-Ph sample were reduced in intensities compared to other treated samples. Such reduction can be correlated with the induced changes in the crystalline structure and molecular arrangement of the inorganic/organic components of the sample, leading to alterations in the vibrational modes and intensities of the functional groups observed in spectroscopic analyses, in consistent with XRD data (Fig. 4). However, some NB-Ph functional groups was not only reduced in intensity, but also shifted in frequency from ≈ 1429 to 1462 cm^−1^, but still ascribed to carbonate group.

#### Microstructural characteristics

**Figure 6:**
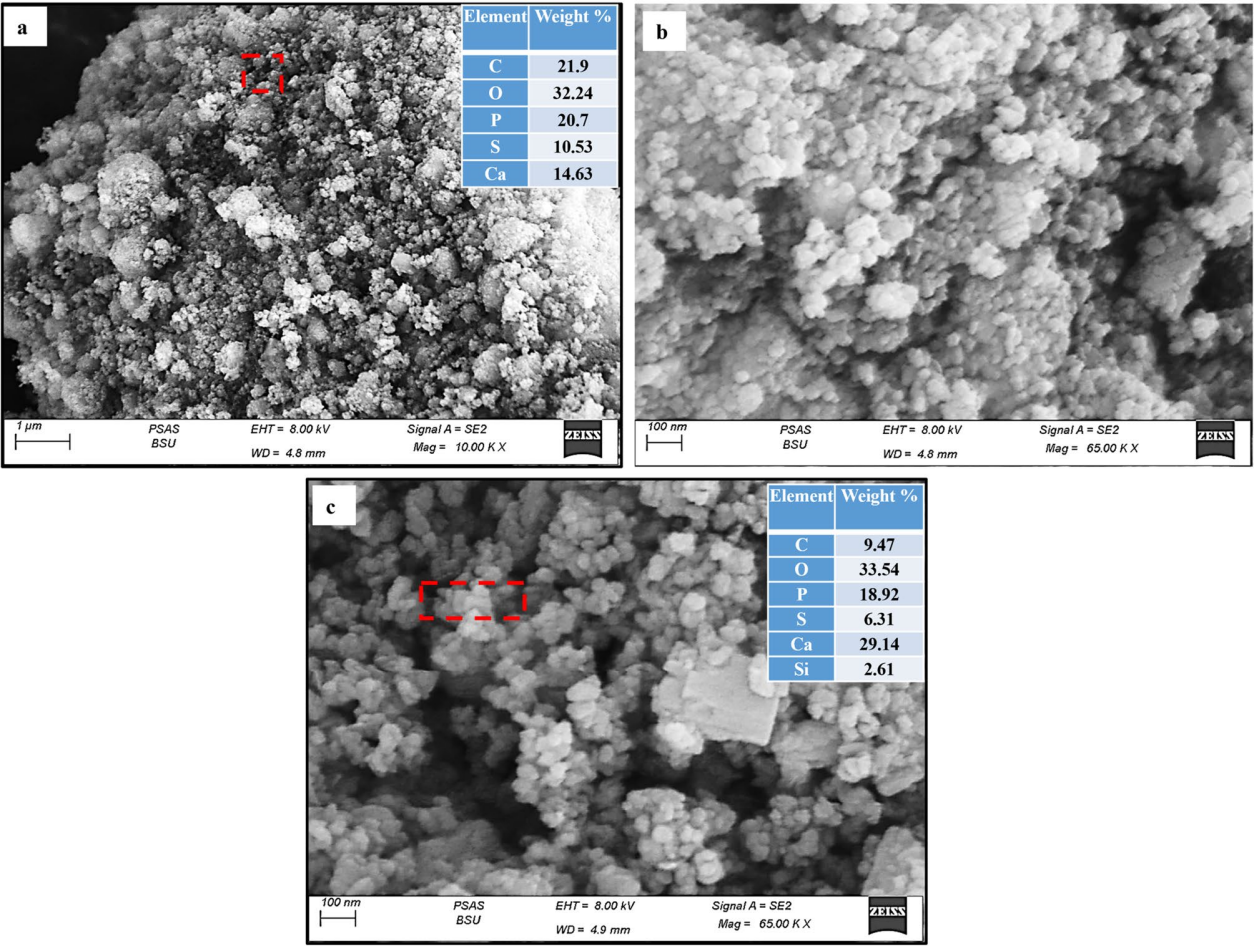
SEM images and EDX of the precursor black phosphate (RB-Ph) (**a**,**b**) and its modified derivative, calcination at 550 ℃/4 h (CB-Ph), (**c**).

**Figure 7:**
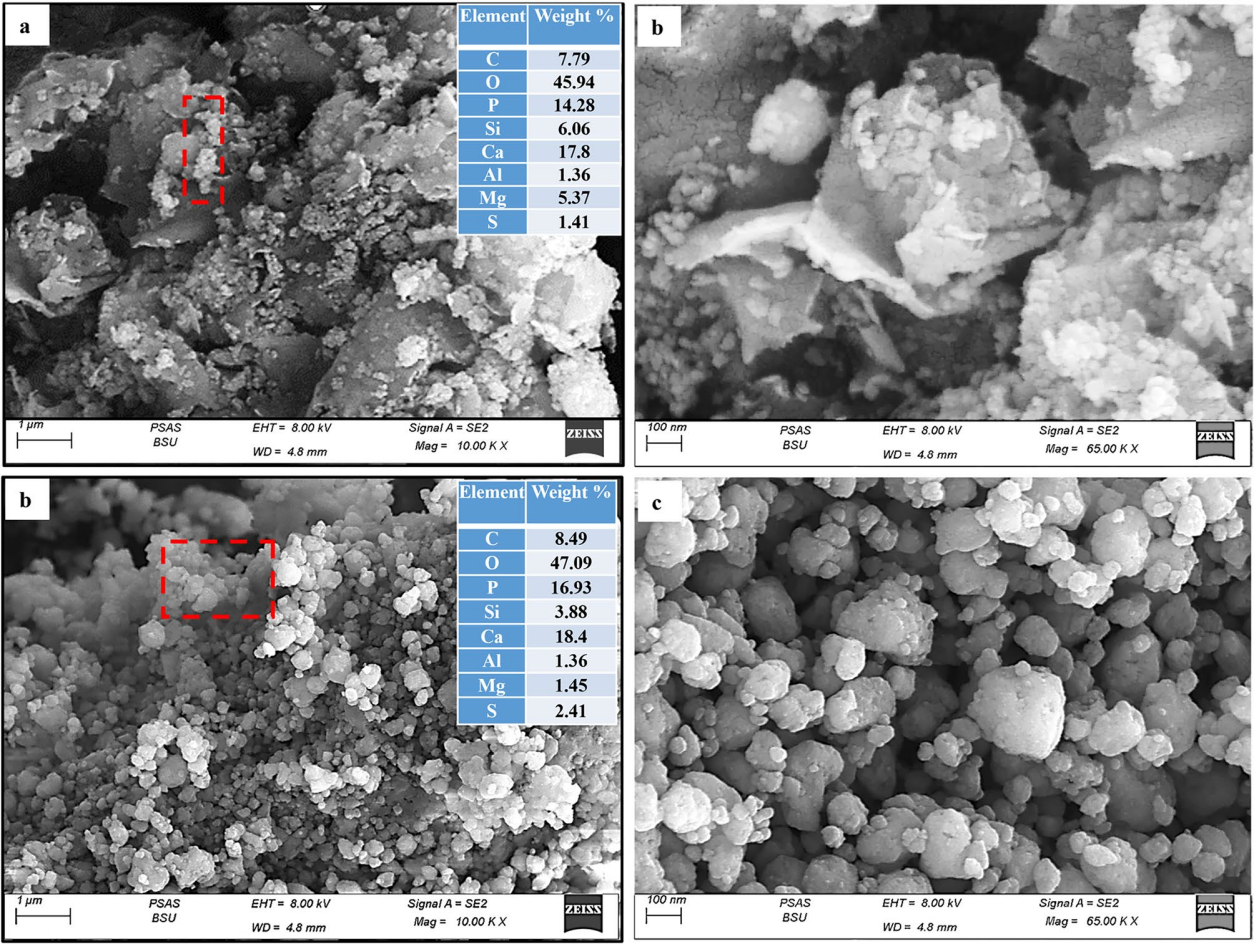
SEM images and EDX of HB-Ph (30% hydrogen peroxide) (**a**,**b**) and NB-Ph (intensive grinding to nano-sizes) samples (**c**,**d**).

The morphology and elemental analysis of the studied phosphorite samples (RB-Ph, CB-Ph, HB-Ph, and NB-Ph) were investigated by SEM and EDX, respectively (Fig. 6a–c and Fig. 7 a–d). The SEM micrographs revealed the inhomogeneity of the RB-Ph sample on the level of grain sizes and shapes (Fig. 6a, b). Whereas the EDX analysis confirmed the high content of C and S, as a sign upon the high OM in the RB-Ph sample (Fig. 6a). But, with calcination at 550 ℃/4 h (CB-Ph), the homogeneity in grain sizes and shapes was approximately improved with slight increase in grain sizes due to the experienced recrystallization process^60,80^ (Fig. 6c). Such homogeneity was interrupted with the occasional presence of some phyllosilicate grains of illite with marked flaky nature^40^ (Fig. 6c). Also, the calcination process contributed to an observable reduction in C and S contents in accordance with the oxidation of the associating OM of the pristine sample during the calcination process^47^. On the contrary, the chemical treatment by H_2_O_2_ (30%), resulted in inhomogeneous morphology in HB-Ph compared to the precursor one (Fig. 7a, b). This inhomogeneous nature could be correlated with the prevalence of curly flakes of the phyllosilicate illite to the limit of rosette morphology formation^81^ (Fig. 7a, b). Additionally, the EDX analysis demonstrated a noticeable decrease in C and S in accordance with the decomposition/oxidation of the OM by H_2_O_2_ (Fig. 7a). Also, the remarkable presence of Mg in the EDX analysis reflects the survived dolomite after the experienced chemical treatment by H_2_O_2_, matching with XRD data (Fig. 4). Furthermore, the impact of intensive grinding on the pristine phosphorite was demonstrated by spherical to semi-spherical grains of phosphatic and non-phosphatic components that vary in grain sizes from 200 to 10 nm (Fig. 7c, d). Some of these fine components may agglomerate together to produce larger particles in correlation with the hydrous nature of phosphatic and some of the non-phosphatic components as was assured by the high LOI content (16.47 wt.%) in the XRF data (Table 2). Also, the disappearance of flaky gains of the clay mineral (illite) affirms the complete structure destruction of these grains with intensive grinding. Moreover, the EDX analysis also illustrates a decline of both C and S contents in NB-Ph compared to the pristine RB-Ph. This could be correlated to the decomposition of the associating OM with grinding to nano-sizes (Fig. 7c). However, the presence of both Mg and Si confirms the presence of dolomite and silicates in NB-Ph sample, matching with XRD and XRF data (Fig. 4 & Table 2).

#### Organic and inorganic components

The total organic carbon (TOC), total inorganic carbon (TIC) and total sulfur (TS) of the pristine phosphorite sample (RB-Ph) before and after calcination (CB-Ph), H_2_O_2_ (30%) treatment (CB-Ph) and intensive ball-mill grinding (NB-Ph) were investigated and complied in Table 3. It was revealed that the calcination of the raw sample at 550 ℃/4 h resulted in a remarkable reduction of the TOC and TIC from 0.543 to 0.097% and 1.627 to 1.052%, orderly. This reduction was correlated with the decomposition of the associated OM^47^ and some of the carbonate minerals (dolomite), respectively. This decomposition results in the release of volatile gases such as carbon dioxide (CO_2_) and water vapor from these organic and inorganic components. Whereas the slight reduction of the TS by calcination from 5.286% in the RB-Ph to 4.768% in CB-Ph sample (Table 3), could be correlated with the decomposition/oxidation of pyrite^82^, matching with XRD results (Fig. 4). It’s important to note that the observed reduction in TS may not solely be attributed to pyrite oxidation, but, also due to the decomposition of organic compounds containing sulfur, such as sulfides or organic sulfates, and their conversion into gaseous sulfur compounds (e.g., sulfur dioxide, SO_2_), which escape from the CB-Ph matrix^83^. Also, the depletion of the TOC (0.209%) in the HB-Ph sample compared to RB-Ph, could be correlated with the decomposition of the associated OM with H_2_O_2_ (30%) treatment, releasing carbon dioxide (CO_2_) and water vapor^84,85^. Conversely, the TIC (5.065%) in the HB-Ph sample displayed a noticeable increase compared to RB-Ph (Table 3). This could be assigned to the prevalence of dolomite that survived from the H_2_O_2_ attacks due to its high chemical resistance to chemical alteration, in agreement with XRD data. Such dolomite, the in organic carbon source, prevalence in concentration was also enforced by the combined effects of OM oxidation and carbon dioxide release. Also, the intensive abatement of TS (0.643%) with H_2_O_2_ treatment probably was correlated not only with pyrite decomposition, but also due to the oxidation of organic compounds containing sulfur by such strong oxidizing agent, releasing SO_2_ from the HB-Ph^86^. Finally, with intensive grinding of RB-Ph to nano-sizes (NB-Ph sample), both TOC (0.410%) and TS (2.669%) were slightly and moderately reduced, orderly, in comparison with RB-Ph (Table 3). The breakdown or fragmentation of the incorporated OM during the experienced mechanical grinding and its accompanying heat of friction, resulted in an increase of surface exposure to air. This triggered the oxidation or decomposition of these matters and the evaporation of their volatile components, leading to such reduction in TOC value in the NB-Ph. Similarly, the moderate reduction of TS could be correlated with the destruction of pyrite structure and OM oxidation/decomposition during the milling process, aligning with XRD data. These collaborative processes facilitated the release of sulfur in form of SO_2_ and hence the reduction of TS with grinding in NB-Ph. On the contrary, the TIC content (2.936%) was surprisingly enhanced with grinding compared to the pristine sample (Table 3). During the grinding process, the mechanical forces that caused the breakdown/decomposition of organic matter and dolomite of the pristine sample lead to the release of both carbon dioxide (CO_2_) and water vapor (H_2_O) into the semi-closed grinding system that was marked with high vapor pressure. These released gases probably reacted with each other, leading to the formation of carbonic acid (H_2_CO_3_), which can further dissociate into bicarbonate ions (HCO^3−^). These bicarbonates probably combined with other cations released from the decomposition of other minerals such as potassium (K^+^) from illite, to form amorphous bicarbonate salts that lack the long-range order required to be detected in the XRD pattern. The presence of these bicarbonate salts was confirmed in both FT-IR spectra (carbonate group at 1462 cm^−1^) and LOI (16.46 wt.%) of the XRF data.

**Table 3:** Total carbon (organic & inorganic) and total sulfur of the precursor black phosphate (RB-Ph) in comparison with its modified derivatives, CB-Ph (calcinated at 550 ℃/4 h), HB-Ph (30% hydrogen peroxide treated) and NB-Ph (intensively ground to nano-sizes).

Sample	Total carbon (TC%)	Total organic carbon (TOC%)	Total inorganic carbon (TIC%)	Total sulfur (TS%)
RB-Ph	2.170	0.543	1.627	5.286
CB-Ph	1.150	0.097	1.052	4.768
HB-Ph	5.275	0.209	5.065	0.643
NB-Ph	3.346	0.410	2.936	2.669

#### Geometrical characteristics

**Figure 8:**
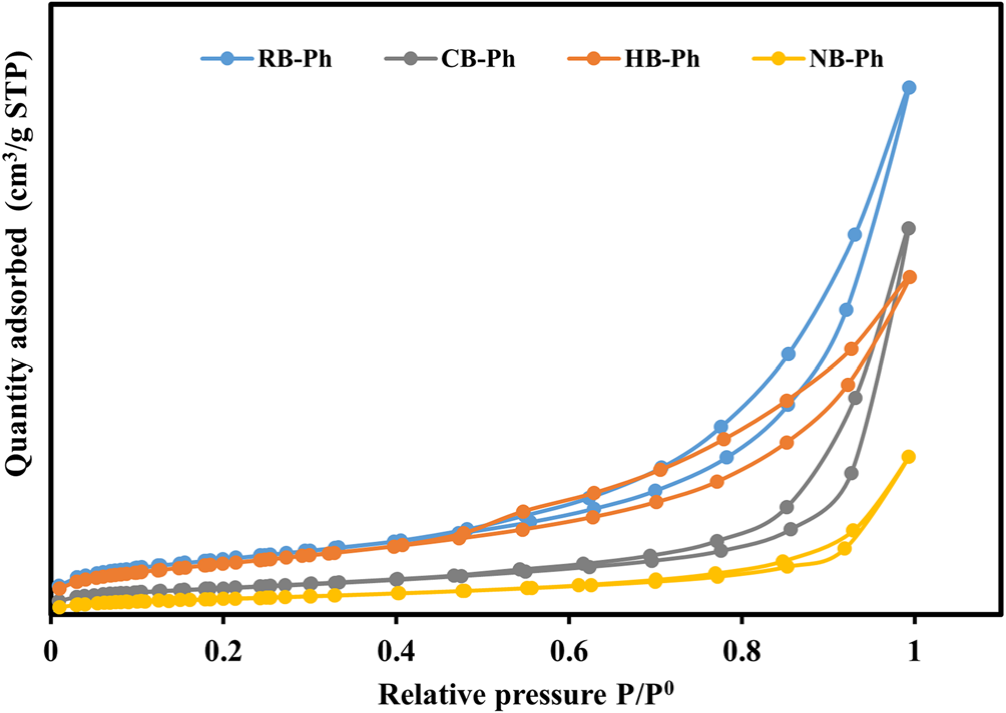
N_2_ adsorption–desorption isotherms of the precursor black phosphate (RB-Ph) in comparison with those of its modified derivatives, calcination at 550 ℃/4 h (CB-Ph), 30% hydrogen peroxide (HB-Ph) and intensive grinding to nano-sizes (NB-Ph).

The N_2_ adsorption/desorption isotherms of RB-Ph and its modified derivatives (CB-Ph, HB-Ph, and NB-Ph) can be classified as Type III isotherms (Fig. 8)^87^, with typical H_3_ hysteresis loops due to capillary condensation on the surface of mesoporous (2–50 nm)^88,89^. These isotherms indicate monolayer followed by multi-layer adsorption; the approximately flat regions at low and medium P/P^0^ in all isotherms is correlated with N_2_ monolayer adsorption^90,91^. Whereas at high P/P^0^, the N_2_ adsorption was converted into multi-layer by the investigated samples. The progressive tightening of the hysteresis loops of the modified derivatives with countable preference with NB-Ph, demonstrates the supremacy of the mesopores. However, the incapacity to attain the equilibrium state of N_2_ adsorption by any of the addressed samples, infers the broad variability in their pore diameters. Aligning with N_2_ isotherm outcomes, the geometrical parameters of the studied samples (Table 4) displayed that the intensive grinding and calcination processes resulted in a deep reduction of S_BET_ and total pore volume** (**V_t_) of the NB-Ph (7.60 m^2^/g and 0.03 cm^3^/g) and CB-Ph (12.40 m^2^/g and 0.07 cm^3^/g) samples. In opposition, the H_2_O_2_ treatment resulted in a minor reduction in S_BET_ (24.30 m^2^/g) of HB-Ph compared to RB-Ph (26.20 m^2^/g). Regarding NB-Ph, the deviation from the fact stating that “the lower the particle sizes, the greater the surface area” might be ascribed to the higher rate of agglomeration with intensive grinding to nano-size as a consequence of the high LOI content of this sample that exceeded 16 wt.%^89,92–94^. Similarly, with calcination at 550 ℃/4 h, the decomposition of OM by oxidation and the escaping of H_2_O vapor, CO_2_ and other volatiles probably led to pores collapse and hence the noticeable reduction in both S_BET_ and V_t_ of CB-Ph compared to RB-Ph^95^. Additionally, the increase in grain sizes that accompanied the experienced recrystallization process with calcination could also justify such S_BET_ and V_t_ reduction, aligning with the SEM results (Fig. 6c). Conversely, the decomposition of associating OM with H_2_O_2_ contributed to the evacuation of the blocked and semi-blocked pores with these matters by oxidation that approximately preserved the S_BET_ (24.3 m^2^/g) close to the pristine sample. However, the collapse of the walls between neighboring mesopores pores resulted in the retrogradation of the overall pore volume of HB-Ph via their partial re-blocking with prevailing dolomite rhombs in agreement with XRD data. As well, the penetrative ability of H_2_O_2_ as a powerful activating agent to produce new micro-pores, especially upon the surface of the flaky particles, side by side with the original meso-pores, could rationalize the recorded average pore diameter, Dp (11.03 nm) that represent the lowest value among all the investigated samples (Table 4).

**Table 4:** Textural parameters of the precursor black phosphate (RB-Ph) sample obtained from N_2_ adsorption–desorption isotherms in comparison with its modified derivatives, CB-Ph (calcinated at 550 ℃/4 h), HB-Ph (30% hydrogen peroxide treated) and NB-Ph (intensively ground to nano-sizes).

Sample	Surface area (m^2^/g)	Total pore volume (cm^3^/g)	Average pore diameter (nm)
	(S_BET_)	V_t_	D_p_
RB-Ph	26.20	0.10	16.01
CB-Ph	12.40	0.07	24.40
HB-Ph	24.30	0.06	11.04
NB-Ph	7.60	0.03	16.40

### Solubility studies

#### Impact of acetic acid concentration

**Figure 9:**
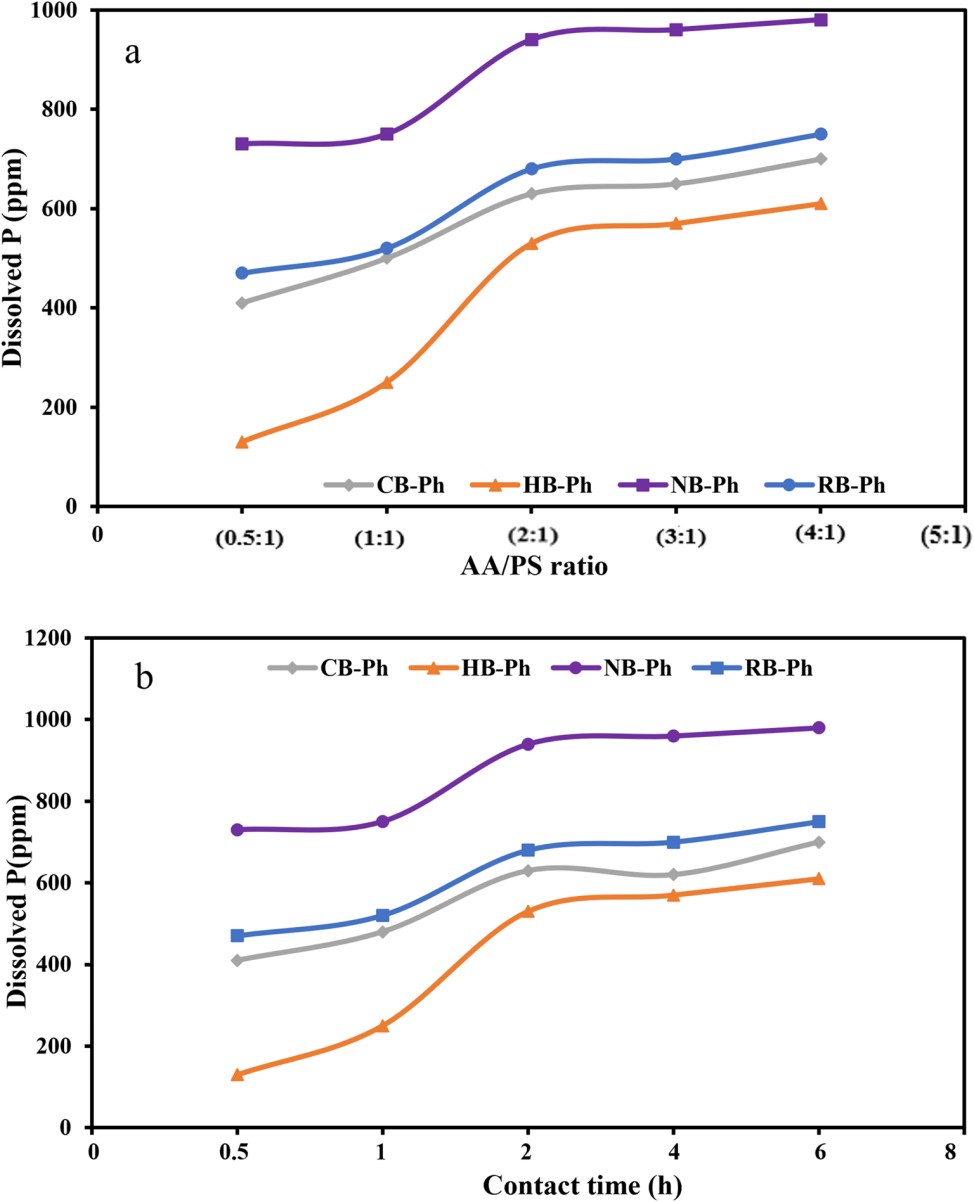
Effects of acetic acid (AA) concentration (**a**) and retention time (**b**) on phosphorus (P) dissolution of the precursor black phosphate (RB-Ph) in comparison with its modified derivatives, calcination at 550 ℃/4 h (CB-Ph), 30% hydrogen peroxide (HB-Ph) and intensive grinding to nano-sizes (NB-Ph).

The effect of acetic acid (AA) concentration upon P dissolution from the prepared phosphorite samples, PS (RB-Ph, CB-Ph, HB-Ph and NB-Ph) at various liquid/solid ratios (AA/PS, w/w) ranging from 0.5:1 to 4:1, was investigated. These investigations were conducted at a fixed PS dose of 1.5 g, shaking speed of 200 rpm/2 h and 25 ml of DW at room temperature (Fig. 9a & Table 1). It was demonstrated that P dissolution increases as the acid concentration rises to a particular level; the dissolution rate was decelerated beyond 2:1 ratio for all addressed samples, i.e., the dissolution rate became insignificant (Fig. 9a). This observation aligns with the findings of other reported data that revealed that the P dissolution rates increase as pH decreases, i.e., increasing the acidity of the medium^96,97^. In general, the proton (H^+^) and ligand (carboxylic group) of organic acid-promote the dissolution processes of P. But in monodentate coordinated organic ligands (such as acetic acid), the carboxylic group effect on the P dissolution is minimal to nonexistent^98^. The protonation of P–O group regulates such dissolution process^99,100^. With the inspection of Fig. 9a, it was revealed that the P dissolution rate from the RB-Ph sample was the highest compared to the other treated samples with calcination and H_2_O_2_. Conversely, the particles diameter (i.e., nano-size) had a conspicuous role in the dissolution process of P as the highest values were recorded in NB-Ph sample, aligning with other reported data^101^.

Although the remarkable P release pattern from thermally treated phosphates that was observed and discussed in several previous investigations^102^, the environmental trade-offs of this high-energy process that provide a critical perspective on its practical application were less emphasized in these studies. Investigations of these drawbacks, such as high energy requirements and emissions, although they were not within the scope of the current study but are important considerations for practical application of this processing technique. Similarly, the enhanced P release via chemical treatment with H_2_O_2_as a viable method for increasing phosphorus availability, was also reported by several studies^103^. However, the environmental considerations of residual H_2_O_2_ in soil and its impact on soil microbiota, are areas that were less covered before, and warrant further research to develop environmentally safe protocols. On the other hand, aligning with the current study, mechanical grinding investigations reported improved nutrient release from finely ground mineral phosphates^104^. But the current study further revealed that this method also mitigates the negative environmental impacts associated with excessive use of synthetic fertilizers, a consideration not fully explored in previous works. However, grinding can sometimes lead to issues such as dust generation and increased energy consumption.

Unlike several reported data^105,106^, it was revealed that OM had a deep and conspicuous impact on the solubility of phosphorus (P); more labile or easily decomposable OM may have transient effects on P solubility. This probably was accomplished through mechanisms such as complexation or the release of organic acids that accompanied OM decomposition by the applied acetic acid, increasing the availability of P for dissolution. Similarly, the OM decomposition most likely allows the release of the adsorbed P by the binding sites on the surface of OM to the solution^107^. On the contrary, the severe reduction of OM, either by calcination or H_2_O_2_ treatments, i.e., the driving factor that was enhancing P solubility was approximately eliminated, led to a decrease in P solubility. However, the drop in P_2_O_5_ wt.% content in the HB-Ph (21.06 wt.%) after H_2_O_2_ treatment cannot be neglected as another intervening factor for the reduction of soluble P during the AA leaching process (Fig. 9a). Conversely, the slight reduction in TOC of the NB-Ph sample (0.410%) coupled with the nano- particle size can collectively justify the remarkable dissolution rate of phosphorous that exceeded 700 ppm for all applied acid ratios although the previously mentioned slight reduction in the P_2_O_5_ wt.% content (22.34 wt.%) with intensive milling process (Table 2). Also, the amorphous nature of the phosphatic-components that lack a well-defined crystalline structure and often more reactive than crystalline counterparts, aligning with XRD data, explain the displayed P dissolution rate of NB-Ph through a greater exposure to the acid solution. This facilitated faster dissolution and higher phosphorus concentrations in the solution. In other words, the lack of a well-defined crystalline structure also means that there are fewer constraints on the dissolution process, allowing easier access to the phosphorus components and hence their subsequent dissolution^108,109^.

Considering the obtained adequate results and for economic/environmental reasons, the 2:1 ratio was nominated for the conduction of the subsequent experiments.

#### Impact of applied retention time

The impact of different retention times (0.5, 1, 2, 4 and 6 h) on P dissolution from the investigated PS (RB-Ph, CB-Ph, HB-Ph and NB-Ph), using a fixed concentration of acetic acid, AA was invasively investigated (Fig. 9b). These leaching experiments were conducted at AA/PS ratio of 2:1, w/w, shaking speed of 200 rpm, and 25 ml DW at room temperature (Table 1). It was displayed that raising the contact time from 0.5 to 2 h was accompanied by an observable increase in the dissolution rate of P for all the investigated samples (Fig. 9b). However, beyond 2 h of retention time, the P dissolution rate was insignificant, signifying that equilibrium state was attained. This was attributed to the consumption of hydrogen ions in solution with time^110–113^. Furthermore, the P dissolution rate followed the following trend for the studied samples over the investigated range of contact time: NB-Ph (730–980 ppm) > RB-Ph (470–710 ppm) > CB-Ph (410–650 ppm) > HB-Ph (130–580 ppm). This remarkable dissolution rate of NB-Ph then RB-Ph in the second rank, reflects the very important role played not only by the particle size of the PS/amorphous nature but also their OM contents, with some preference of the formers. Moreover, the drop in both P_2_O_5_ wt.% content (21.06 wt.%) and TOC (0.209%), as well as the crystalline nature of the HB-Ph with H_2_O_2_ treatment, can be accounted as intervening factors for the reduction of soluble P during the AA leaching process.

#### Regeneration studies

**Figure 10:**
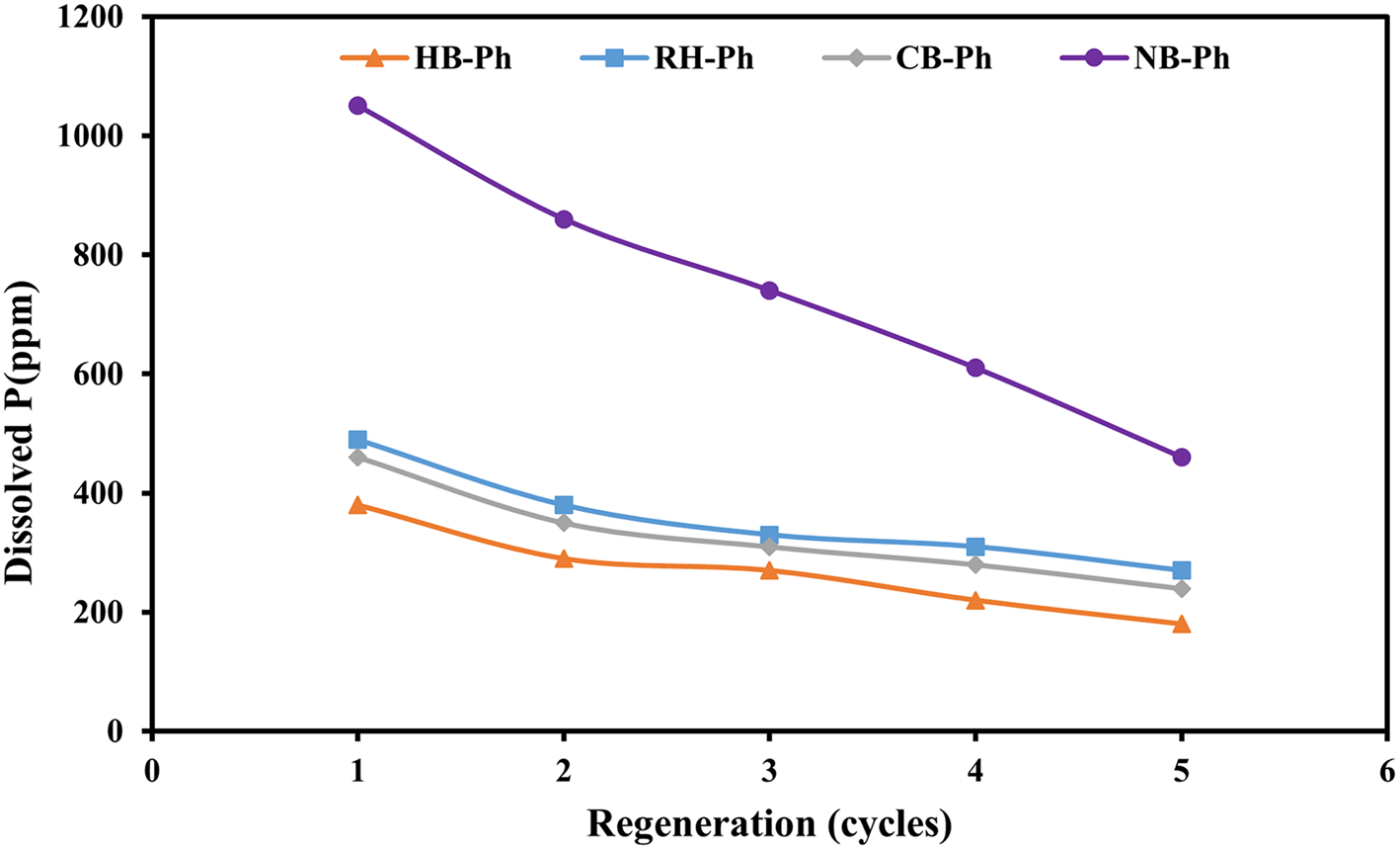
Regeneration studies elaborate the magnitude of phosphorus (P) dissolution rate along five leaching cycles of the precursor black phosphate (RB-Ph) in comparison with its modified derivatives, calcination at 550 ℃/4 h (CB-Ph), 30% hydrogen peroxide (HB-Ph) and intensive grinding to nano-sizes (NB-Ph) samples.

In order to evaluate the potential amount of dissolved P through the leaching processes out of RB-Ph, CB-Ph, HB-Ph, and NB-Ph, separately, several leaching cycles were conducted, using a fixed concentration of AA that achieves a ratio of AA/PS, 2:1 w/w as depicted in Fig. 10, Table 1. Unlike NB-Ph, the dissolution rate of P at the first leaching cycle of RB-Ph, CB-Ph and HB-Ph samples in comparison with the results of the equivalent leaching experiments of the previously investigated parameters (acid concentration and retention time), was obviously reduced (Fig. 10). This could be attributed to the decline of the solid–liquid interface in accordance with agglomeration/stacking of these PS particles within the AA solution, preventing the hydrogen ions of the acetic acid from having the opportunity to contact the surfaces of the unreacted particles^114^. The immunity of the NB-Ph against P dissolution declines at the 1st leaching cycle could be justified by the high diffusion of its particles within the AA solution. This led to an increase in the solid–liquid interface and hence the P dissolution rate was improved compared to the obtained results of the previously mentioned equivalent experiments (Fig. 10). Moreover, a gradual decrease in P dissolution for all the addressed PS was observed with each applied leaching cycle (Fig. 10). However, the dissolution persistency till the 5th leaching cycle with a preference of NB-Ph sample over the others, confirms the continuity of P solubility in accordance with particle size reduction during the successive leaching cycles^115,116^.

## Conclusion

The outputs of the current work can be compiled in the following bullets:

- To highlight the impact of OM content on P solubility during AA leaching, the mineralogical and morphological characteristics of phosphatic and non-phosphatic components of the black phosphate (RB-Ph), before and after physical (calcination at 550 ℃/4 h, CB-Ph), chemical (30% H_2_O_2,_ HB-Ph) and mechanical (intensive grinding to nanoscale, NB-Ph) treatments, were carefully investigated.
- Intensive grinding to nanoscale (NB-Ph) resulted in amorphous phosphatic and non-phosphatic components, significantly enhancing P dissolution rates (730–980 ppm) compared to other treatment protocols despite its noticeable reduction in P_2_O_5_ (22.34 wt.%).
- Despite the high TOC (0.543%) and P_2_O_5_ (30.51 wt.%) contents of RB-Ph, the P dissolution rate (470–750 ppm) with AA was sufficiently adequate compared to NB-Ph that has a slightly and highly reduced TOC (0.410%) and P_2_O_5_ (22.34 wt.%) contents, orderly, as a response of intensive grinding.
- Calcination at 550 ℃/4 h (CB-Ph) also influenced P solubility, demonstrating comparable dissolution rates (410–700 ppm) despite its changes in TOC (0.097%) and P_2_O_5_ (30.36 wt.%) contents, as well as the noticeable variation in crystallinity.
- Treatment of RB-Ph with H_2_O_2_ (30%) not only enhanced the intensity of the dolomite and the overall crystallinity, but also reduced the P_2_O_5_ (21.60 wt.%) & TOC (0.209%) contents and diminished P solubility of the HB-Ph (130–610 ppm) compared to the other addressed samples.
- Unlike NB-Ph, the P dissolution rate of RB-Ph, CB-Ph and HB-Ph at the first leaching cycle of regeneration studies in comparison with the equivalent leaching experiments of the investigated parameters (acid concentration and retention time), was obviously reduced. This was ascribed to its particles agglomeration/stacking within the AA solution, preventing hydrogen ions from proper contact with the surfaces of these unreacted particles.
- The improvement of P dissolution of NB-Ph at the first leaching cycle (1150 ppm) could be justified by the high diffusion rate of its particles within AA solution, increasing the solid–liquid interface compared to the obtained results of the equivalent leaching experiments of the other parameters (≈ 940 ppm).
- The P dissolution persistency till the fifth leaching cycle with preference of NB-Ph sample over the others, aligns with the fact of particle size reduction during the successive leaching cycles.
- Finally, mechanical grinding emerges as a promising treatment to maximize the agronomic potential of black phosphate deposits, paving the way for sustainable practices that benefit both agricultural productivity and environmental health.

## Environmental and practical implications of applied treatments on phosphorus availability in soil

The various treatments applied to black phosphate (RB-Ph)—including physical (calcination), chemical (H_2_O_2_ treatment), and mechanical (intensive grinding)—may have significant environmental and practical implications for phosphorus (P) availability in soil. Calcination, while effective in altering mineral phases and reducing organic content, may have environmental drawbacks due to the high energy consumption and potential release of greenhouse gases. Chemical treatments, such as H_2_O_2_, can improve P release by oxidizing organic matter, but the environmental impacts of residual chemicals and their interaction with soil microbiota require careful consideration. Conversely, mechanical grinding, which enhances P solubility by creating amorphous structures and increasing surface area, offers a practical solution for improving nutrient availability in soils, potentially reducing the need for synthetic fertilizers and mitigating phosphorus runoff into water bodies. Therefore, understanding these implications helps in selecting sustainable treatment protocols that enhance phosphorus recovery while minimizing environmental footprints, thus supporting the development of efficient and eco-friendly agricultural practices and nutrient management strategies.

### Future research directions and applications

Future research should focus on understanding the long-term effects of these treatments on phosphorus availability and soil health, conducting field trials to validate lab findings, and exploring environmentally sustainable treatment protocols. Practical applications of these findings include optimizing nutrient management strategies in agriculture and enhancing phosphorus recovery from black phosphate deposits, contributing to improved agricultural productivity and environmental stewardship.

- Finally, investigating the mechanisms of enhanced phosphorus solubility, optimizing treatment protocols for efficiency and sustainability, and conducting field trials to validate laboratory findings are the main future research directions of the current study.

## Data Availability

I declare that the data supporting the findings of this study are available within the paper and its Supplementary Information files, and data will be made available.
